# Comparison of the Kato-Katz, Wet Mount, and Formol-Ether Concentration Diagnostic Techniques for Intestinal Helminth Infections in Ethiopia

**DOI:** 10.5402/2013/180439

**Published:** 2012-10-22

**Authors:** Mengistu Endris, Zinaye Tekeste, Wossenseged Lemma, Afework Kassu

**Affiliations:** School of Biomedical and Laboratory Sciences, College of Medicine and Health Sciences, University of Gondar, P.O. Box 196, Gondar, Ethiopia

## Abstract

*Objective*. The aim of this study was to evaluate the operational characteristics (sensitivity and negative predictive value (NPV)) of wet mount, formol-ether concentration (FEC), and Kato-Katz techniques for the determination of intestinal parasitic infections. *Method*. A total of 354 faecal specimens were collected from students in Northwest Ethiopia and screened with Kato-Katz, wet mount, and FEC for the presence of intestinal parasitic infection. Since a gold standard test is not available for detection of intestinal parasites, the combined results from the three methods were used as diagnostic gold standard. *Result*. The prevalences of intestinal parasites using the single wet mount, FEC, and Kato-Katz thick smear techniques were 38.4%, 57.1%, and 59%, respectively. Taking the combined results of three techniques as a standard test for intestinal parasitic infection, the sensitivity and negative predictive value of Kato-Katz is 81.0% (confidence interval (CI) = 0.793–0.810) and 66.2% (CI = 0.63–0.622), respectively. The FEC detected 56 negative samples that were positive by the gold standard, indicating 78.3% (CI = 0.766–0.783) and 63.2% (CI = 0.603–63) sensitivity and NPV, respectively. Furthermore, Kato-Katz detects 113 cases that were negative by a single wet mount. The *κ* agreement between the wet mount and Kato-Katz methods for the diagnosis of *Ascaris lumbricoides* and hookworm was substantial (*κ* = 0.61 for *Ascaris lumbricoides*, *κ* = 0.65 for hookworm).

## 1. Background

Intestinal parasitic infections are among the most common infections worldwide. It is estimated that 3.5 billion people are affected, and 450 million are ill as a result of these infections, the majority being children [1]. In Ethiopia, intestinal parasitic infection is the second most predominant cause of outpatient morbidity. However, there are difficulties in estimating the exact burden of parasitic infections in the country [2]. Although there are several factors that make estimating the number and burden of intestinal parasitic infections difficult, lack of accurate diagnostic tools is the major one [3–9].

Although several diagnostic methods such as Kato-Katz and Formol-Ether Concentration (FEC) techniques are available, direct wet mount is the commonly used as a reliable diagnosis method for the diagnosis of intestinal parasitic infections generally in Africa and particularly in Ethiopia [10–13]. However, low sensitivity of the direct wet mount technique has been reported in the detection of low-intensity infection elsewhere [14]. This shows that the use of direct wet mount as a confirmatory test will significantly increase misdiagnosis of intestinal helminth infections. The reliable diagnosis of intestinal parasitic infections requires a more rapid, easy, and sensitive method. Therefore, this study aimed to evaluate the operational characteristics (sensitivity and negative predictive value (NPV)) of wet mount, FEC, and Kato-Katz techniques in intestinal parasitic infection endemic locality of Ethiopia. 

## 2. Methods

### 2.1. Study Design and Area

A cross-sectional study was conducted in Atse Fasil Elementary School from March 10 to June 30, 2008 in Northwest Ethiopia, latitude of 12.55 (12°33′0 N), a longitude of 37.43 (37°25′32 E), and an elevation of 1,994 meters above sea level [15].

### 2.2. Study Population

A total of 354 students were included in the study. Students who had no history of antihelmenthic drug administration in the two weeks prior to screening, absence of any other serious chronic infection, and had ability to give stool samples were included in the study.

### 2.3. Stool Collection and Analysis

Students were provided with plastic stool cups and asked to bring approximately 3 gm of fresh stool of their own. Approximately 41.7 mg and 20 mg of the stool specimen were used to prepare a single Kato-Katz thick smear and wet mount preparation, respectively. One gram of stool sample was analyzed by FEC technique [16]. All the samples were processed in the temporally established laboratory except the Kato-Katz smear count of the common helminthes, which was done at the laboratory of the University of Gondar. The egg per gram of faeces (EPGs) counts for hookworm were done immediately in the temporary laboratory. The Kato-thick smear counts for all the common helminthes except for hookworm were done at the laboratory of University of Gondar.

### 2.4. Quality Control

Before starting the actual work, quality of reagents and instruments was checked by experienced laboratory technicians. The specimens were also checked for serial number, quantity, and procedures of collection. 

To eliminate observer bias, each stool sample was examined immediately in the temporary laboratory by two experienced laboratory technicians. The technicians were not told about the health and other status of the study participants. In cases where the results were discordant, a third expert reader was used. The results of the third expert reader were considered the final result.

### 2.5. Data Analysis

Since a “gold” standard test is not available for detection of intestinal parasites, the operational characteristics (sensitivity and NPV) were estimated using the combined results from the three methods as diagnostic “gold” standard [17, 18].

Data were analyzed using SPSS version 16 and JavaStat software. Sensitivities, NPV, and kappa were determined for the various tests to evaluate their operational characteristics. *P* values <0.05 were considered statistically significant.

### 2.6. Ethical Considerations

The study protocol was reviewed and approved by the Ethical Review Committee of the Department of Medical Laboratory Technology, University of Gondar. Written informed consent was obtained from all study participants and mothers/caretakers of students under 18 who participated in the study after explaining the purpose and objective of the study. Students who were positive for intestinal parasites were treated based on the recommended drug regimen at Azezo Clinic, Gondar.

## 3. Result

A total of 354 students aged 5–19 years participated in the study; 146 (53.9%) and 125 (46.1%) were males and females, respectively. 

### 3.1. Prevalence

The prevalences of intestinal parasites using single wet mount, FEC, and Kato-Katz thick smear techniques were 38.4%, 57.1%, and 59%, respectively (Table 1). The detection rate when two techniques used at a time was 69.8% (for Kato-thick and FEC), 61% (for wet mount and FEC), and 68% (for wet mount and Kato-thick smear technique). The detection rate was 72.9% (258/354) when all the three techniques were used together. The overall prevalence of *Schistosoma mansoni*, *Ascaris lumbricoides, Trichuris trichiura, *and hookworm infections was 43.5%, 28.8%, 18.1%, and 8.2%, respectively (Table 2).

### 3.2. Sensitivity and NPV

 Among the 354 study participants diagnosed 209 were found to be positive while 145 were negative by Kato-Katz. The sensitivity and NPV of Kato-Katz are 81.0% (confidence interval (CI) = 0.793–0.810) and 66.2% (CI = 0.63–0.622), respectively (Table 1). The FEC detected 56 negative samples that were positive by the gold standard, indicating 78.3% (CI = 0.766–0.783) and 63.2% (CI = 0.603–63) sensitivity and NPV, respectively (Table 1).

### 3.3. Agreement in Test Results

As compared to single FEC, single Kato-Katz detected 53, 83, 56, and 95 cases of *Ascaris lumbricoides, Schistosoma mansoni*, *Trichuris trichiura, *and hook worm, respectively (Table 3), indicating substantial (*κ* = 0.80), moderate (*κ* = 0.58), and moderate (*κ* = 0.59) *κ* agreement between the FEC and Kato-Katz for the diagnosis of *Ascaris lumbricoides*, *Schistosoma mansoni *and *Trichuris trichiura*, respectively (Table 3). Furthermore, Kato-Katz detects 113 cases that were negative by a single wet mount (Table 4). The *κ* agreement between the wet mount and Kato-Katz methods for the diagnosis of *Ascaris lumbricoides *and hook worm was substantial (*κ* = 0.61 for *Ascaris lumbricoides*, *κ* = 0.65 for hookworm) (Table 4).

## 4. Discussion

A single Kato-Katz had significantly lower detection capacity than the FEC method in diagnosing hookworm. This poor performance of the Kato-Katz in detecting hookworm infection is explained by the following facts. First, hookworm has lower egg laying capacity, more likely to be missed by Kato-Katz. Second, hookworm eggs disappear due to glycerin when long time delays occur between Kato-Katz smear preparation and microscopic examination [19]. Furthermore, unlike FEC, small amount of fecal material is processed in Kato-Katz technique. The chance of detecting hookworm infection by Kato-Katz from small amount of faecal material is suggested to be lower [20, 21]. Therefore, small amount of fecal material used in Kato-Katz technique may be the reason for lower detection capacity of Kato-Katz.

The finding of the present study showed that, as compared to the Kato-Katz and FEC techniques, direct wet mount exhibited very low sensitivity for the detection of *Ascaris lumbricoides*, *Schistosoma mansoni*, *Trichuris trichiura *and hookworm. The use of direct wet mount alone as an indicator of intestinal parasitic infections is also suggested to be insufficient by other studies [22]. However, in most laboratories in Ethiopia, the direct wet mount is the preferred stool parasitological detection technique. This shows that, since the use of direct wet mount as a confirmatory test will significantly increase misdiagnosis of intestinal helminth infections, the use of another diagnosing method is mandatory to decrease the consequences caused in the community due to intestinal helminth infections.

As compared to FEC and direct wet mount techniques, the Kato-Katz exhibited high sensitivity for the detection of *Schistosoma mansoni*, *Trichuris trichiura, *and *Ascaris lumbricoides*. This shows that the use of the Kato-Katz as a confirmatory test for *Schistosoma mansoni*, *Trichuris trichiura, *and *Ascaris lumbricoides *will reduce the morbidity and mortality caused by these parasites by reducing misdiagnosis. However, lack of previous similar study makes difficulty in making rigorous discussion on this finding.

In conclusion, the present study revealed that Kato-Katz technique and FEC methods showed a better sensitivity than the traditional direct wet mount method. Therefore, the employment of FEC technique as a confirmatory test in routine laboratory examination of stool and Kato-Katz in epidemiological studies will significantly aid in accurate determination and management of parasitic infections in the community.

## Figures and Tables

**Table 1:** Wet mount, Kato-Katz, and FEC techniques results compared to the gold standard from Atse Fasil General Elementary School, Northwest Ethiopia, March 10–June 30, 2008.

Method	Result	Gold standard		Total (%)	NPV	95% CI	Sensitivity	95% CI
		Positive (%)	Negative (%)					
Wet mount	Positive	136 (38.4)	0	136 (38.4)	44.0%	0.42–0.44	52.7%	0.51–0.53
	Negative	122 (34.5)	96 (27.1)	218 (61.6)				
FEC	Positive	202 (57.1)	0	202 (57.1)	63.2%	0.60–0.63	78.3%	0.76–0.78
	Negative	56 (15.8)	96 (27.1)	152 (42.9)				
Kato-Katz	Positive	209 (59.0)	0	209 (59.0)	66.2%	0.63–0.62	81.0%	0.79–0.81
	Negative	49 (13.8)	96 (27.1)	145 (41.0)				

FEC: formol-ether concentration, NPV: negative predictive value.

**Table 2:** Wet mount, Kato-Katz, and FEC techniques results compared to the gold standard by species of common helminthes from students of Atse Fasil General Elementary School, Northwest Ethiopia, March 10–June 30, 2008.

Parasite	Technique	Infected students no. (%)	NPV (95% CI)	Sensitivity (95% CI)
*S. mansoni *	Gold standard	154 (43.5)		
	Kato katz	148 (41.8)	97.1% (0.95–0.97)	96.1% (0.94–0.96)
	FEC	90 (25.4)	75.8% (0.74–0.75)	58.4% (0.55–0.58)
	Wet mount	34 (9.6)	62.5% (0.61–0.62)	22.1% (0.194–0.221)

*A. lumbricoides *	Gold standard	102 (28.8)		
	Kato-Katz	95 (26.8)	97.3% (0.95–0.97)	93.1% (0.89–0.93)
	FEC	83 (23.4)	93.0% (0.92–0.93)	81.4% (0.77–0.81)
	Wet mount	53 (15)	83.7% (0.82–0.83)	52.0% (0.48–0.52)

*T. trichiura *	Gold standard	64 (18.1)		
	Kato-Katz	58 (16.4)	98.0% (0.97–0.98)	90.6% (0.85–0.91)
	FEC	37 (10.5)	91.5% (0.90–0.92)	57.8% (0.52–0.58)
	Wet mount	8 (2.3)	83.8% (0.83–0.84)	12.5% (0.07–0.12)

Hook worm	Gold standard	29 (8.2)		
	Kato-Katz	20 (5.6)	97.3% (0.96–0.97)	69.0% (0.57–0.69)
	FEC	21 (5.9)	97.6% (0.97–0.98)	72.4% (0.60–0.72)
	Wet mount	11 (3.1)	94.8% (0.94–0.95)	37.9% (0.27–0.38)

FEC: formol-ether concentration, NPV: negative predictive value.

**Table 3:** Agreement between a single Kato-Katz and FEC technique for the diagnosis of each intestinal parasite in students from Atse Fasil General Elementary School, Northwest Ethiopia, March 10–June 30, 2008.

Parasite	FEC	Kato-Katz		Total	*κ* agreement (*P*)
		Positive	Negative		
*S. mansoni *	Positive	85	5	85	0.58 (*P* < 0.001)
	Negative	62	202	264	

	Total	147	207	354	

*A. lumbricoides *	Positive	75	8	83	0.80 (*P* < 0.001)
	Negative	18	253	271	

	Total	93	261	354	

*T. trichiura *	Positive	31	6	37	0.59 (*P* < 0.001)
	Negative	28	289	317	

	Total	59	295	354	

Hook worm	Positive	12	9	21	0.57 (*P* < 0.001)
	Negative	7	326	333	

	Total	19	335	354	

FEC: formol-ether concentration.

**Table 4:** Agreement between a single Kato-Katz and wet mount for the diagnosis of each intestinal parasite in students from Atse Fasil General Elementary School, Northwest Ethiopia, March 10–June 30, 2008.

Parasite	Wet mount	Kato-Katz		Total	*κ* agreement (*P*)
		Positive	Negative		
*S. mansoni *	Positive	33	1	34	0.25 (*P* < 0.001)
	Negative	114	206	320	

	Total	147	207	354	

*A. lumbricoides *	Positive	50	3	53	0.61 (*P* < 0.001)
	Negative	43	258	301	

	Total	93	261	354	

*T. trichiura *	Positive	8	0	8	0.21 (*P* < 0.001)
	Negative	51	295	346	

	Total	59	295	354	

Hook worm	Positive	10	1	11	0.65 (*P* < 0.001)
	Negative	9	334	343	

	Total	19	335	354	
